# Patent foramen ovale closure versus medical therapy for stroke prevention: A systematic review and meta-analysis of randomized controlled trials

**DOI:** 10.12688/f1000research.13444.2

**Published:** 2018-09-11

**Authors:** Jenny Chi Ling Lai, Gary Tse, William K.K. Wu, Mengqi Gong, George Bazoukis, Wing Tak Wong, Sunny Hei Wong, Konstantinos Lampropoulos, Adrian Baranchuk, Lap Ah Tse, Yunlong Xia, Guangping Li, Martin C.S. Wong, Yat Sun Chan, Nan Mu, Mei Dong, Tong Liu, International Health Informatics Study (IHIS) Network

**Affiliations:** 1 Department of Medicine and Therapeutics, Faculty of Medicine, Chinese University of Hong Kong, Hong Kong, Hong Kong; 2 Li Ka Shing Institute of Health Sciences, Faculty of Medicine, Chinese University of Hong Kong, Hong Kong, Hong Kong; 3 Department of Anaesthesia and Intensive Care, State Key Laboratory of Digestive Disease, Chinese University of Hong Kong, Hong Kong, Hong Kong; 4 Tianjin Key Laboratory of Ionic-Molecular Function of Cardiovascular disease, Department of Cardiology, Tianjin Institute of Cardiology, The Second Hospital of Tianjin Medical University, Tianjin, 300211, China; 5 Second Department of Cardiology, Evangelismos General Hospital of Athens, Athens, Greece; 6 School of Life Sciences, Chinese University of Hong Kong, Hong Kong, Hong Kong; 7 State Key Laboratory of Agrobiotechnology, Chinese University of Hong Kong, Hong Kong, Hong Kong; 8 Division of Cardiology, Kingston General Hospital, Queen’s University, Kingston, ON, Canada; 9 JC School of Public Health and Primary Care, Chinese University of Hong Kong, Hong Kong, Hong Kong; 10 Department of Cardiovascular Medicine, First Affiliated Hospital of Dalian Medical University, Dalian, China; 11 Department of Gynecology and Obstetrics, Yantai Yuhuangding Hospital Affiliated to Qingdao University, Yantai, Shandong, 264000, China

**Keywords:** Patent foramen ovale, PFO closure, stroke, medical therapy

## Abstract

**Background:** Previous randomized trials on patent foramen ovale (PFO) closure versus medical therapy for stroke prevention were inconclusive. Recently, two new randomized trials and new findings from an extended follow-up of a previous trial have been published on this topic. We conducted a systematic review and meta-analysis of randomized trials comparing PFO closure with medical therapy for stroke prevention.

**Methods: **PubMed and Cochrane Library were searched until 16 ^th^ September 2017.  The following search terms were used for PubMed: "patent foramen ovale" AND (stroke OR embolism) and "randomized" AND "Trial". For Cochrane Library, the following terms were used: "patent foramen ovale" AND "closure" AND (stroke OR embolism).

**Results: **A total of 91 and 55 entries were retrieved from each database using our search strategy respectively, of which six studies on five trials met the inclusion criteria. This meta-analysis included 1829 patients in the PFO closure arm (mean age: 45.3 years; 54% male) and 1972 patients in the medical therapy arm (mean age: 45.1 years; 51% male). The median follow-up duration was 50 ± 30 months. When compared to medical therapy, PFO closure significantly reduced primary endpoint events with a risk ratio [RR] of 0.60 (95% CI: 0.44-0.83, P < 0.0001; *I* ^2^: 15%). It also reduced stroke (RR: 0.50, 95% CI: 0.35-0.73, P < 0.0001; *I* ^2^: 32%) despite increasing the risk of atrial fibrillation/flutter (RR: 1.90, 95% CI: 1.23-2.93, P < 0.01; *I* ^2^: 43%). However, it did not reduce transient ischemic accident events (0.75; 95% CI: 0.51-1.10, P = 0.14; *I* ^2^: 0%), all-cause bleeding (RR: 0.89; 95% CI: 0.44-1.78, P = 0.74; *I* ^2^: 51%) or gastrointestinal complications (RR: 0.92; 95% CI: 0.32-2.70, P = 0.88; *I* ^2^: 0%).

**Conclusions:** PFO closure significantly reduces risk of stroke when compared to medical treatment and should therefore be considered for stroke prevention in PFO patients.

## Introduction

The association between the presence of a patent foramen ovale (PFO) and cryptogenic stroke has been established by previous case-control studies ^1^. However, whether PFO closure is effective in reducing stroke events when compared to medical therapy is controversial. Three randomized trials, the STARFlex Septal Closure in Patients with a Stroke and/or Transient Ischemic Attack due to Presumed Paradoxical Embolism through a PFO (CLOSURE I) ^2^, the Clinical Trial Comparing Percutaneous Closure of PFO Using the Amplatzer PFO Occluder with Medical Treatment in Patients with Cryptogenic Embolism (PC) ^3^ and the Randomized Evaluation of Recurrent Stroke Comparing PFO Closure to Established Current Standard of Care Treatment (RESPECT) ^4^, were conducted. All of these trials showed numerically fewer events in the primary intention-to-treat analysis, but this did not reach statistical significance. Recently, two trials have focused on this issue. Firstly, the PFO Closure or Anticoagulants versus Antiplatelet Therapy to Prevent Stroke Recurrent (CLOSE) trial evaluated PFO closure or anticoagulation against antiplatelet therapy, with a primary endpoint of fatal or non-fatal stroke ^5^. Secondly, the Gore Helex septal occlude and antiplatelet medical management of reduction of recurrent stroke or imaging-confirmed transient ischemic attack in patients with PFO (REDUCE) trial compared PFO closure to antiplatelet therapy only, with a primary endpoint of ischemic stroke, new ischemic stroke or silent brain infarction, demonstrating significant reductions in these events compared to antiplatelet therapy ^6^. Moreover, long-term data of the RESPECT trial were recently published ^7^. Given these new findings, we conducted a systematic review and meta-analysis of these randomized trials to evaluate the benefits and complication rates in PFO closure versus medical therapy.

## Methods

### Search strategy, inclusion and exclusion criteria

The meta-analysis was performed according to the Preferred Reporting Items for Systematic Reviews and Meta-Analyses statement ( PRISMA; a completed checklist can be found in Supplementary File 1). PubMed and Cochrane Library were searched for randomized trials that compared the efficacy in stroke prevention of PFO closure with that of medical therapy. The following search terms were used for PubMed: "patent foramen ovale" AND (stroke OR embolism) and "randomized" AND "Trial". For Cochrane Library, the following terms were used: "patent foramen ovale" AND "closure" AND (stroke OR embolism). The search period was from the beginning of the databases through to 16 ^th^ September 2017, with no language restrictions.

The following inclusion criteria were applied: i) the design was a randomized trial in humans, ii) the study compared stroke outcomes for PFO closure versus medical therapy. Quality assessment of randomized controlled trials was performed using the Cochrane Risk Assessment Tool ( Supplementary Figure 1 and Supplementary Figure 2).

### Data extraction and statistical analysis

Data from the different studies were entered in Microsoft Excel (2016 Version). All publications extracted from the search strategy were assessed for compliance with the inclusion criteria. In this meta-analysis, the extracted data elements consisted of: i) trial name; ii) follow-up duration; iii) quality score; and iv) characteristics of the population, including sample size, sex and age. Two reviewers (GT and MG) independently reviewed each included study and disagreements were resolved by adjudication with input from a third reviewer (TL).

The number of events for: i) primary endpoint, ii) stroke, iii) transient ischemic attack (TIA), iv) all-cause bleeding complications, v) gastrointestinal complications [bleeding, ulceration, ulcer perforation], vi) short-term AF or AFL, vii) long-term AF or AFL, viii) venous thromboembolism, were identified and extracted independently by each reviewer from each trial. The Comprehensive Meta-Analysis Software (Version 2) was used for subsequent meta-analyses and statistical analyses. The event rates (events per patient-year) were used to calculate rate ratios for each study, which were pooled in subsequent meta-analyses.

Heterogeneity across studies was assessed using the *I ^2^* statistic from the standard chi-square test, which describes the percentage of the variability in the effect estimates resulting from heterogeneity. *I ^2^* > 50% was considered to reflect significant statistical heterogeneity and in such cases the random-effects model was used. To identify the origin of the heterogeneity, sensitivity analysis excluding one study at a time. Funnel plots showing standard errors against the logarithms of the odds ratio were constructed. Begg and Mazumdar rank correlation test was used to test for publication bias and the Egger’s test were used to detect publication bias.

## Results

**Figure 1.**
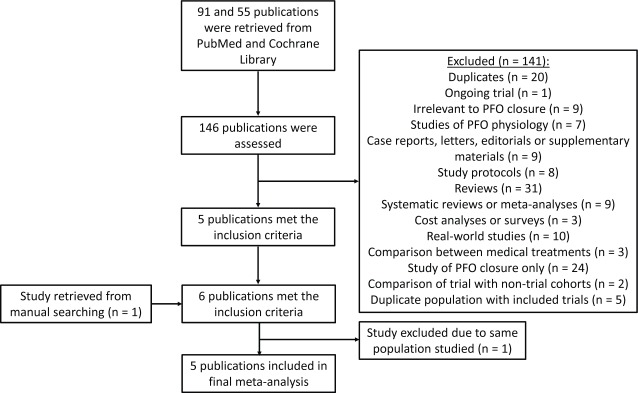
Flowchart of the database search and study selection process.

A flow diagram detailing the above search terms with inclusion and exclusion criteria is depicted in Figure 1. A total of 91 and 55 studies were retrieved from PubMed and Cochrane Library, respectively. Of these, six studies met our inclusion criteria ^3– 8^. These were based on the following trials: CLOSURE I, PC, CLOSE, RESPECT and REDUCE. The original publication on the RESPECT trial was excluded because an update on longer term results was recently published ^7^. Therefore, a total of five studies were included in this meta-analysis ^2– 6^. The baseline characteristics of these studies are listed in Supplementary Table 1. This meta-analysis included 1829 patients in the PFO closure arm (mean age: 45.3 years; 54% male; mean follow-up duration 50 ± 30 months) and 1972 patients in the medical therapy arm (mean age: 45.1 years; 51% male; mean follow-up duration 50 ± 31 months). For the meta-analyses, event rates (events per patient-year) were extracted and used to calculate rate ratios. The results of the statistical treatment of this meta-analysis are detailed in Supplementary File 2, with those of sensitivity analyses by the leave-one-out method described in Supplementary Figure 3– Supplementary Figure 8. Funnel plots plotting standard errors against the logarithms of the risk ratios are shown in Supplementary Figure 9– Supplementary Figure 14. In all of the analyses, Begg and Mazumdar rank correlation test suggested no significant publication bias (P > 0.05) and the Egger’s test demonstrated no asymmetry (P > 0.05).

### PFO closure and primary endpoint(s), stroke and TIA events

**Figure 2.**
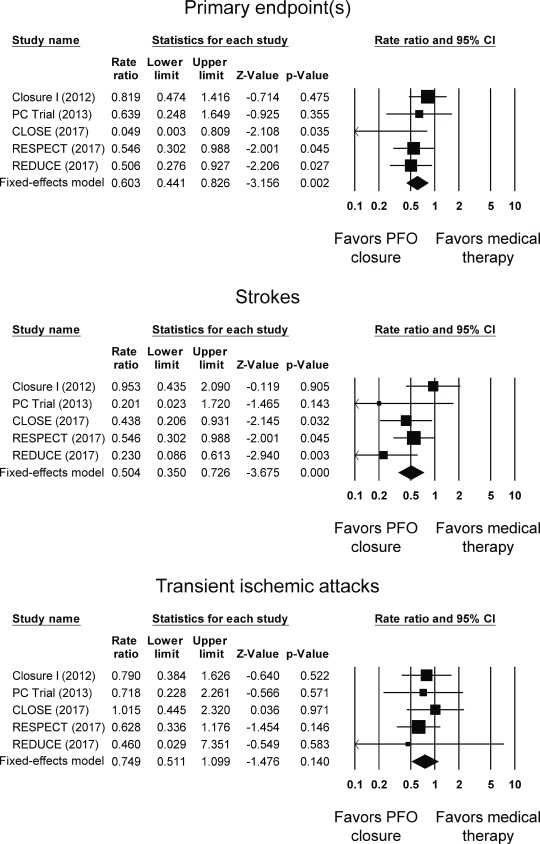
Rate ratios for primary endpoint(s) (top), strokes (middle) and transient ischemic attacks (TIAs; bottom) in patent foramen ovale (PFO) closure versus medical therapy.

All five trials compared the primary endpoints in PFO closure versus medical therapy. The different trials used slightly different primary endpoints ( Supplementary Table 2), as follows: 1) CLOSURE I trial: stroke, TIA, 30-day mortality, neurology-related death, 2) PC trial: stroke, TIA, death, peripheral embolism, 3) CLOSE trial: fatal or non-fatal stroke, 4) RESPECT trial: nonfatal ischemic stroke, fatal ischemic stroke, or early death after randomization and 5) REDUCE trial: co-primary endpoints of i) ischemic stroke, and ii) new ischemic stroke or silent brain infarction. Among the 1829 subjects who underwent PFO closure, 70 (3.8%) met the primary endpoint ( Supplementary Table 3). By contrast, 112 of the 1972 subjects receiving medical therapy (5.7%) met the primary endpoint. Our meta-analysis shows that PFO closure significantly reduced primary endpoint events when compared to medical therapy with a rate ratio [RR] of 0.60 (95% CI: 0.44-0.83, P < 0.0001; *I* ^2^: 15%) ( Figure 2, top panel). Using hazard ratios [HR] from the trials produced negligible differences from our event rate analyses (HR: 0.61; 95% CI: 0.45-0.83, P < 0.01; *I* ^2^: 0%).

Subgroup analyses were performed for the primary endpoint based on atrial septal aneurysm and shunt size by pooling hazard ratios from the subgroup analyses of the included studies. PFO closure was not significantly better than medical therapy for patients with an atrial septal aneurysm (HR: 0.46; 95% CI: 0.14-1.60, P = 0.22; *I* ^2^: 62%, Supplementary Figure 15) or without an atrial septal aneurysm (HR: 0.74; 95% CI: 0.47-1.17, P = 0.19; *I* ^2^: 0%, Supplementary Figure 16). By contrast, PFO closure significantly reduced primary endpoint events in patients with large shunt size (HR: 0.27, 95% CI: 0.14-0.54, P < 0.0001; *I* ^2^: 0%, Supplementary Figure 17) but not in those with small shunt size (HR: 0.80, 95% CI: 0.49-1.31; P = 0.38; *I* ^2^: 0%, Supplementary Figure 18)

All five trials compared the stroke events in PFO closure versus medical therapy. The different trials used slightly different primary endpoints, as follows: 1) CLOSURE I trial: acute focal neurological event that is magnet resonance imaging (MRI) positive, regardless of duration of clinical symptoms, or if imaging cannot be performed for confirmation, it was defined as a persistent focal neurological deficit lasting longer than 24 hours; 2) PC trial: any neurologic deficit lasting for >24 hours typically with documentation in MRI or computer tomography (CT); 3) CLOSE trial: sudden onset of focal neurological symptoms with the presence of cerebral infarction in the appropriate territory on brain imaging (CT or MRI), regardless of the duration of the symptoms (less than or greater than 24 hours); 4) RESPECT trial: ischemic stroke was defined as an acute focal neurologic deficit, which was presumed to be due to focal ischemia, and either symptoms that persisted for 24 hours or longer or symptoms that persisted for less than 24 hours but were associated with findings of a new, neuroanatomically relevant, cerebral infarct on MRI or CT; and 5) REDUCE trial: an acute focal neurologic deficit, presumably due to ischemia, that either resulted in clinical symptoms lasting 24 hours or more or was associated with evidence of relevant infarction on MRI or, if MRI could not be performed, CT of the brain. Taken together data from all five trials, stroke occurred in 45 patients (2.5%) in the PFO closure group, but in 102 patients (5.2%) in the medical therapy group. This gave a RR of 0.50 that was statistically significant (95% CI: 0.35-0.73, P < 0.0001; *I* ^2^: 32%) ( Figure 2, middle panel). Using HR from the trials produced negligible differences from our event rate analyses (HR: 0.49, 95% CI: 0.34-0.71, P < 0.01; *I* ^2^: 30%).

TIAs were assessed in all five trials and the various definitions are shown in Supplementary Table 2. These occurred in 44 patients (2.4%) in the PFO closure group and in 67 patients (3.4%) of the medical therapy group ( Supplementary Table 3). There was no statistically significant difference in the RR (0.75; 95% CI: 0.51-1.10, P = 0.14; *I* ^2^: 0%) ( Figure 2, bottom panel). Hazard ratios were available from four trials on TIAs, with a pooled HR of 0.73 (95% CI: 0.49-1.09, P = 0.13; *I* ^2^: 0%).

### Adverse events in PFO closure group compared to medical therapy group

**Figure 3.**
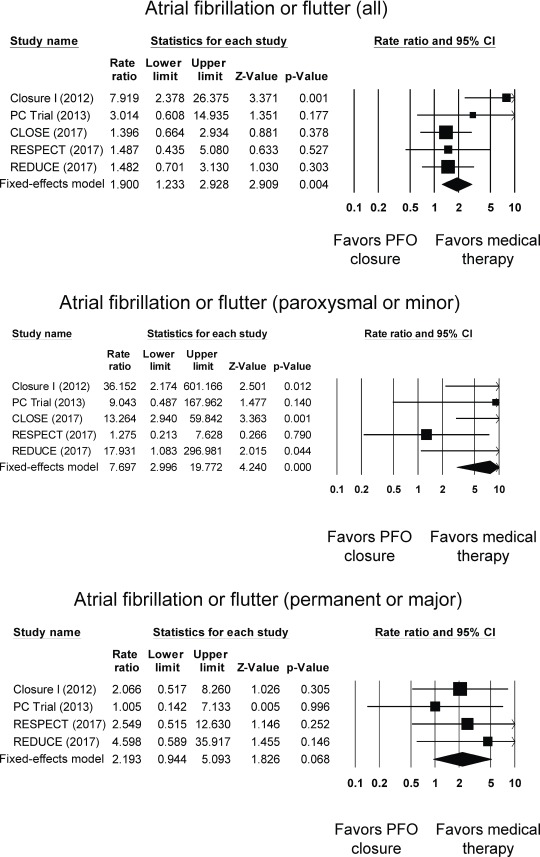
Rate ratios for atrial fibrillation or flutter (all types: top; paroxysmal or minor: middle; permanent or major: bottom) in patent foramen ovale (PFO) closure versus medical therapy.

AF or AFL was detected in 76 patients in the PFO closure group (4.2%) and 37 patients (1.9%) in the medical therapy group ( Supplementary Table 4). These equated to a significant increase in the risk when PFO closure was used (RR: 1.90, 95% CI: 1.23-2.93, P < 0.01; *I* ^2^: 43%) ( Figure 3, top panel). Subgroup analysis was performed for the type of AF or AFL by dividing the episodes into i) paroxysmal or minor, and ii) permanent or major [as defined by the individual trials]. This revealed that most of the episodes were only paroxysmal or minor for the PFO group (3.0%) when compared to the medical therapy group (0.6%) (RR: 7.70, 95% CI: 2.30-19.77; P < 0.0001; *I* ^2^: 32%) ( Figure 3, middle panel). Permanent or serious AF or AFL occurred in 1.3% in the PFO closure group compared to 0.4% in the medical therapy group without significance between these groups (RR: 2.19, 95% CI: 0.94-5.01; P = 0.07; *I* ^2^: 0%) ( Figure 3, bottom panel).

**Figure 4.**
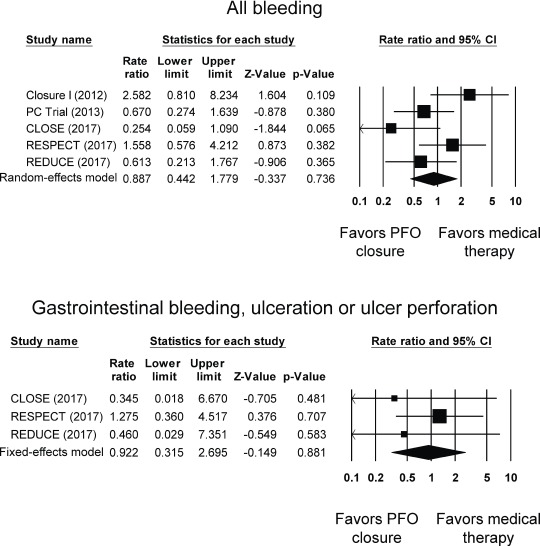
Rate ratios for bleeding (top) and gastrointestinal complications (bottom) in patent foramen ovale (PFO) closure versus medical therapy.

All bleeding complications were counted from the included studies ( Supplementary Table 5). These were comparable between the groups, occurring in 39 (2.1%) of the PFO closure group and 47 (2.4%) in the medical therapy group, with no significant difference between them (RR: 0.89; 95% CI: 0.44-1.78, P = 0.74; *I* ^2^: 51%) ( Figure 4, top panel). Three trials reported gastrointestinal complications of hemorrhage, ulceration or ulcer perforation ( Supplementary Table 5), which occurred in 7 and 8 patients in the PFO closure and medical therapy groups (0.4% for both arms). Therefore, the risk of gastrointestinal complications was not reduced by PFO closure (RR: 0.92; 95% CI: 0.32-2.70, P = 0.88; *I* ^2^: 0%) ( Figure 4, bottom panel). Venous thromboembolism, which comprised events of pulmonary embolism and deep venous thrombosis, were also counted from the included studies ( Supplementary Table 6). Two trials, RESPECT and REDUCE trials, reported venous thromboembolism, which occurred in 20 and 6 patients in the PFO closure and medical therapy groups.

## Discussion

The key findings of this meta-analysis are that, compared to medical therapy, PFO closure significantly reduced primary endpoints by 40% and strokes by 50%, and had comparable risks of TIAs. Nevertheless, these benefits were observed despite a two-fold increase in the risk of AF or AFL in the PFO closure group. No difference in the risks of bleeding or gastrointestinal complications (bleeding, ulceration or ulcer perforation) was observed.

The foramen ovale remains open in about 25% of the healthy population, giving rise to a PFO ^9^. PFO can be asymptomatic, but potentially causes cryptogenic strokes mainly through the mechanism of paradoxical embolization. PFO closure is used either for primary or secondary prevention of stroke. It has been proposed that PFO closure is an effective treatment to prevent recurrent stroke or TIA in patients with cryptogenic stroke if the shunt grade of the PFO is greater than moderate ^10^. Long-term follow-up following percutaneous PFO closure for presumed paradoxical embolism have demonstrated very low recurrence rates ^11, 12^. Eustachian valve, Chiari’s network, medium-large shunt on trans-esophageal echocardiography, hypertension, age and the Essen stroke risk score have been associated with recurrent neurological events ^12– 14^. Medical treatment using antiplatelets or anticoagulants is an acceptable, alternative approach. A recent meta-analysis reported that anticoagulant therapy was more effective than antiplatelet therapy in preventing recurrent stroke and/or TIA, but with a 6-fold greater risk of major bleeding ^15^.

The evidence from real-world studies has also been controversial. A small cohort including 159 patients <55 years old with cryptogenic stroke who received PFO closure or medical therapy did not show a statistically significant difference in the recurrence of ischemic events during a mean follow-up of 51.6 months ^16^. In addition, in another small cohort including 164 patients with PFO and cryptogenic stroke the two groups (PFO closure vs. medical treatment) did not differ in regard to the composite end-point of death, stroke, TIA or peripheral embolism ^17^. Similarly, data of the IPSYS registry, which included 521 patients aged 18–45 years old with cryptogenic stroke and PFO, showed no significant difference neither in composite end-point [ischemic stroke, TIA or peripheral embolism (P=0.285)] nor in brain ischemia (p=0.168) between PFO closure and medical treatment groups ^18^. Additionally, Mirzada and colleagues did not find a difference regarding recurrent stroke or TIA between PFO closure and medical treatment groups ^19^. Most of studies about PFO closure included young patients (<55 years old). A recent study, compared the outcomes of PFO closure between a young (<55 years old) and an old group (>55 years old) of patients ^20^. It was found that in older patients, PFO closure was as safe as in younger patients, but recurrent cerebral ischemia was more frequent and likely this is associated to conditions related to age than to paradoxical embolism ^20^.

Previous meta-analyses of randomized trials have found no statistically significant differences between PFO closure and medical therapy in the prevention of recurrent ischemic stroke ^21– 24^, whilst PFO closure was associated with an increased risk of AF ^21, 24^. By contrast, others have reported some benefits. For example, a patient-level meta-analysis reported that PFO closure reduced recurrent stroke and had a statistically significant effect on the composite of stroke, TIA, and mortality in adjusted analyses ^25^. Moreover, another reported a 50% relative reduction of stroke and/or TIA versus antiplatelet therapy and by 82% relative reduction of major bleeding versus anticoagulant therapy ^15^, whilst another reported that significant reductions in recurrent neurological events in intention-to-treat, per-protocol and as-treated cohorts ^26^. A recent meta-analysis also reported that in comparison with medical treatment, PFO prevents recurrent stroke and TIA ^27^. Further, another recent meta-analysis reported that in patients with PFO and cryptogenic stroke, transcatheter device closure decreases risk of recurrent stroke compared with medical therapy alone ^28^. By contrast, anther meta-analysis reported that PFO reduced the risk of stroke, but not TIA, mortality, major bleeding and increased the risk of AF ^29^. By pooling together data from two additional trials, our meta-analysis provides a firm conclusion that PFO closure produces a statistically significant reduction in the risk of not only primary endpoints, but also that of strokes. On subgroup analysis, we found that PFO closure significantly reduced primary endpoint events in patients with large shunt size, but not in those with small shunt size. Moreover, although PFO closure was no more effective in reducing TIAs when compared to medical therapy, what is important is that stroke, which is responsible for significant morbidity as well as mortality, was prevented. These benefits are also observed in real-world studies. For example, a long-term propensity score-matched comparison of PFO closure with medical therapy showed a mortality benefit ^30^. These data also raise the issue regarding the benefit of primary prevention in cases with high-risk PFO ^31^. Such a simple intervention might indeed be effective in preventing the first stroke event. As such, based on the results of our meta-analysis, it supports the need to primarily prevent high-risk PFO with PFO closure procedures instead of providing medical therapy. This approach is further by the increasing simplicity and success rates of the PFO closure procedure ^32^. Despite the clear benefits, potential complications of PFO closure should be noted. For example, in the RESPECT trial, a significant increase in pulmonary emboli were observed (12 in the PFO closure group, of which 2 are listed as a complication of the procedure, vs. 3 in the control group) and deep venous thromboses (5 and 1, respectively). While the mechanism leading to the increased risk remains unclear, it may be possibly attributable to inappropriate application of groin compression subsequent to PFO intervention. Our meta-analysis also confirmed the increased risk of AF following PFO closure. Its clinical relevance is less certain for two reasons, that 70–90% of these events occurred in the first month and did not persist beyond this timeframe, and stroke incidence was reduced despite this AF occurrence. While the majority of AF occurrences were transient AF, it is not known how much subsequent monitoring these patients underwent or whether they were anticoagulated as a result.

In our meta-analysis of event rates for primary endpoint(s), low levels of heterogeneity were observed, which was probably due to the different definitions used across the trials. For example, two trials, CLOSURE I ^8^ and PC ^3^, included mortality as part of the primary endpoint, whereas the remaining three trials, CLOSE ^5^, RESPECT ^4^ and REDUCE ^6^, included ischemic stroke events but not mortality. The medical therapy offered was similar across the studies, involving anticoagulation, antiplatelet therapy, or both. For the CLOSE trial, the investigators compared antiplatelet therapy with either anticoagulation therapy or PFO closure. For this particular trial, we had pooled data from both anticoagulation and antiplatelet therapy together, and compared the event rates with the PFO closure group. The present meta-analysis also demonstrates the safety of PFO closure when compared to more conservative medical therapy.

### Strengths and limitations of the included trials and this meta-analysis

However, there are some limitations inherent to the trials themselves. Firstly, the crossover rate is substantial. For example, in REDUCE 6.3% of study subjects crossed over from PFO-closure to medical treatment and 6.3% did the opposite. Secondly, actual mechanical and anatomical closure of the PFO is of paramount importance to prevent recurrent paradoxical embolization. The different trials had different degrees of successful closure (e.g. REDUCE trial: 75%; CLOSURE trial: 86%; CLOSE trial: 93%) but these were not related to outcomes. Secondly, the definitions of TIA were different between the trials, which could have contributed to the between-study heterogeneity. Finally, the trials had large proportions of study subjects who were lost to follow-up, withdrew consent and crossed over to the other study arm, leading to uncertainty in the reported event rates.

There are many strengths of this meta-analysis study. It is the largest meta-analysis of randomized trials to date, including more than 3800 participants from five trials. Moreover, the follow-up duration was sufficiently long for events to be detected. Our quality analysis indicated that the studies had a low risk of bias. Low levels of heterogeneity were observed for most of our analyses, including primary endpoints, strokes, TIAs and gastrointestinal complications. These indicate that it was appropriate to pool these studies together. However, several limitations inherent in the present meta-analysis should be noted. Firstly, the meta-analysis of all-cause bleeding across the trials showed a high level of heterogeneity, which may be clinical in nature, especially when different types of bleeding were measured. Secondly, there is an imbalance between events and lost-to-follow-up/withdrawals ^33^. In most trials, the ratio between the two is often in the region of 5 to 1, but is around 0.3 to 1 in the trials included in this meta-analysis. Moreover, publication bias results should be interpreted with caution as publication bias with less than ten studies is not recommended, but the results are presented here for the sake of completeness. Furthermore, there was a moderate degree of heterogeneity for the atrial septal aneurysm vs. no aneurysm comparison. Since our aim was to compare the effectiveness of PFO closure to medical treatment, we did not perform additional analysis by medical group assignment, such as comparing antiplatelet or anticoagulant therapy to PFO closure. The majority of medical management patients were treated with antiplatelet medications, with a minority treated with oral anticoagulation. Since the presumed mechanism of stroke attributable to PFO is paradoxical embolus from venous thrombi, or *in situ* thrombus formation, oral anticoagulants may be more effective for those causes. Where reported in individual trials, stroke outcome events were numerically less when medical management was with oral anticoagulation. Further analyses are needed to compare PFO closure with anticoagulation treatment alone as this was beyond the scope of the current study.

## Conclusions

PFO closure significantly reduces the risk of primary endpoints, strokes, but not TIAs, when compared to medical treatment, despite higher rates of AF or AFL being observed. No differences in bleeding or gastrointestinal complications were detected between the two arms. PFO closure should therefore be considered for prevention of stroke in patients with PFO, especially in those presenting with cryptogenic stroke, who are less than 60 years of age with moderate to severe shunting.

## Data availability

All data required for reproducibility of this study are available from published studies.

## Supplementary material

Supplementary File 1: PRISMA checklist.
