# Supplementary material for: Patent foramen ovale closure versus medical therapy for stroke prevention: A systematic review and meta-analysis of randomized controlled trials
Source: F1000Res. 2018 Sep 11;6:2178. Originally published 2017 Dec 27. [Version 2] doi: 10.12688/f1000research.13444.2 (PMC6137413; doi:10.12688/f1000research.13444.2)
Supplement: Supplementary file 2 [file f1000research-6-17114-s0001.tgz › 3dced615-75d2-4321-ac57-c09f318e795e.pdf]

Supplementary Appendix

Supplementary Figures

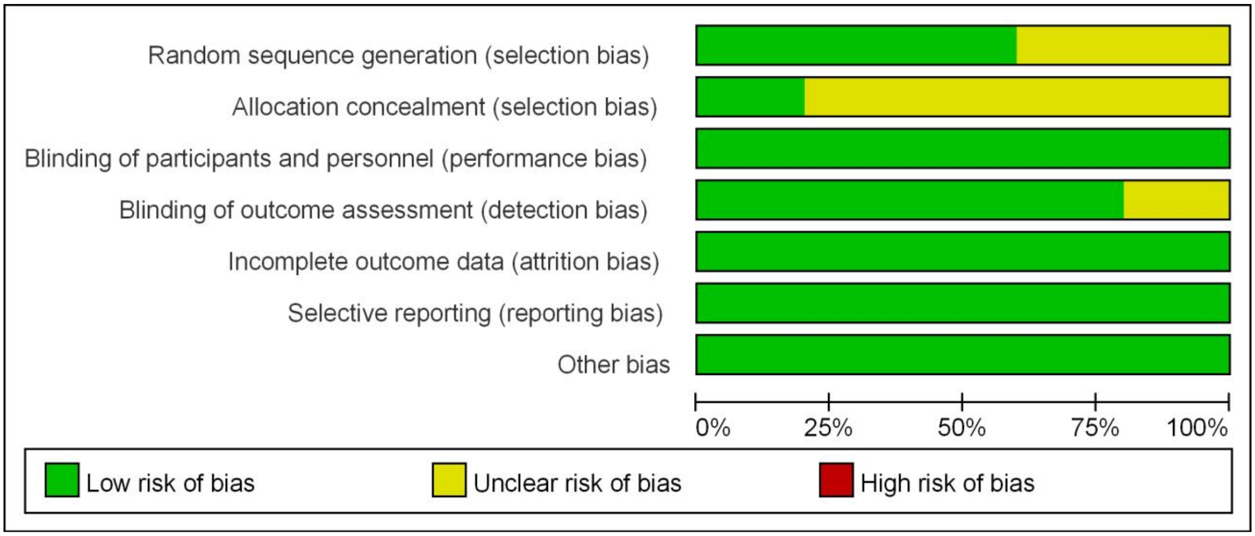

Supplementary Figure 1. Risk of bias graph using the Cochrane Risk Assessment Tool.

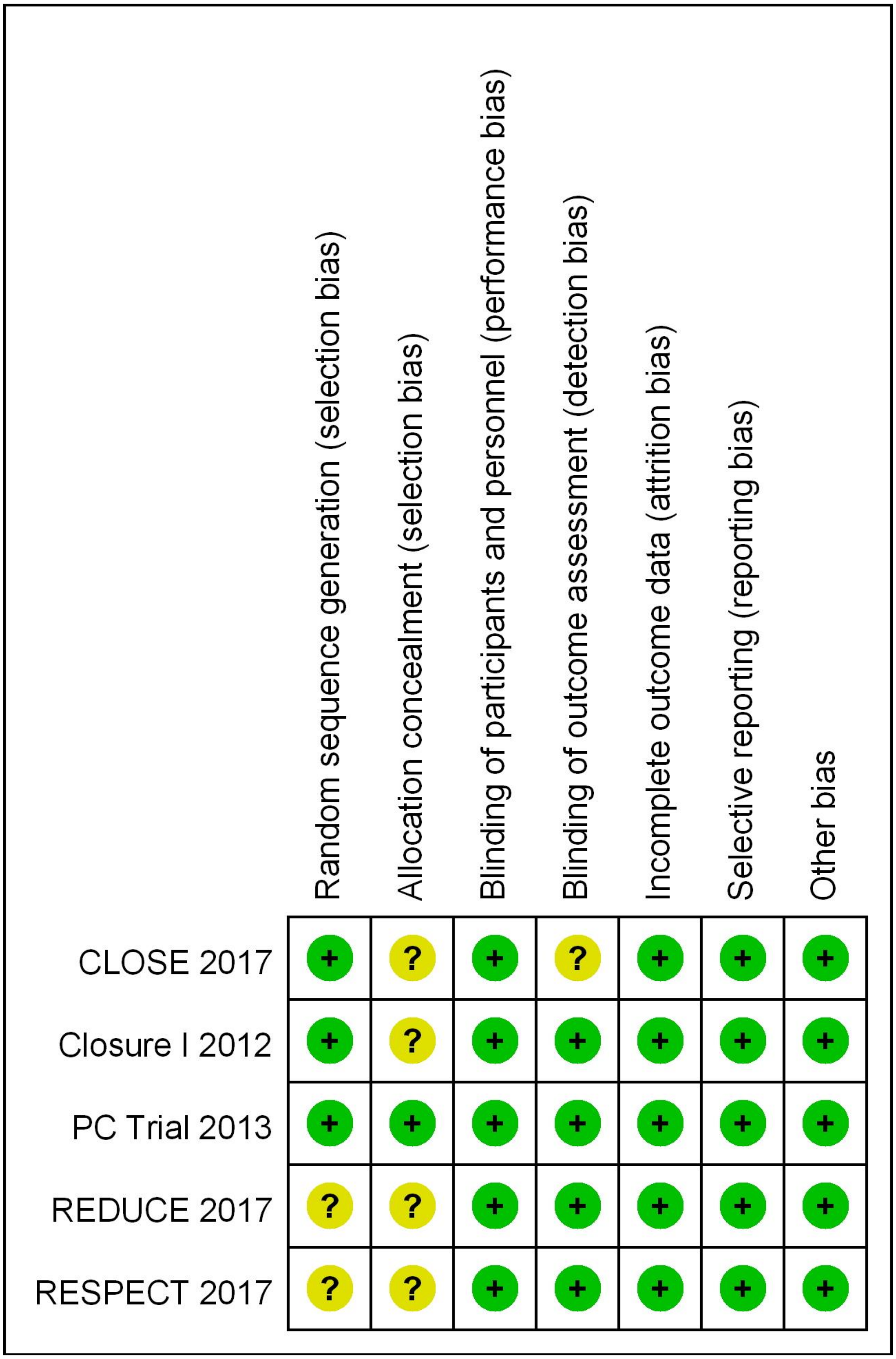

Supplementary Figure 2. Risk of bias summary using the Cochrane Risk Assessment Tool.

Primary endpoint: sensitivity analysis

Study name	Statistics with study removed				
	Point	Lower limit	Upper limit	Z-Value	p-Value
Closure I (2012)	0.51	0.36	0.71	-3.89	0.00
PC Trial (2013)	0.58	0.43	0.78	-3.57	0.00
CLOSE (2017)	0.60	0.45	0.80	-3.48	0.00
RESPECT (2017)	0.57	0.41	0.80	-3.30	0.00
REDUCE (2017)	0.68	0.48	0.97	-2.11	0.04
Fixed-effects model	0.58	0.44	0.78	-3.67	0.00

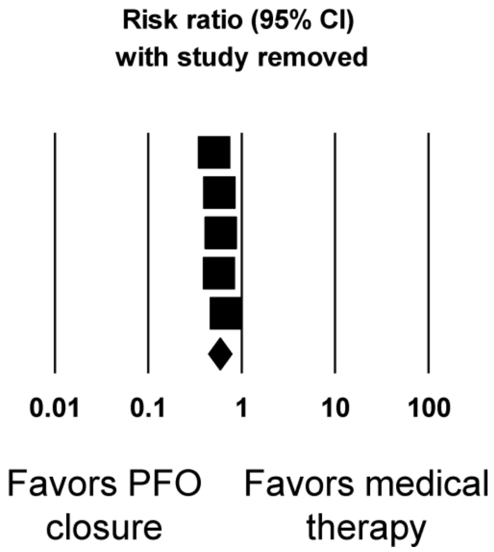

Supplementary Figure 3. Sensitivity analysis for primary endpoint(s).

Strokes: sensitivity analysis

Study name	Statistics with study removed				
	Point	Lower limit	Upper limit	Z-Value	p-Value
Closure I (2012)	0.46	0.31	0.70	-3.72	0.00
PC Trial (2013)	0.56	0.39	0.80	-3.15	0.00
CLOSE (2017)	0.57	0.38	0.86	-2.67	0.01
RESPECT (2017)	0.50	0.32	0.79	-2.99	0.00
REDUCE (2017)	0.61	0.42	0.90	-2.49	0.01
Fixed-effects model	0.54	0.38	0.78	-3.35	0.00

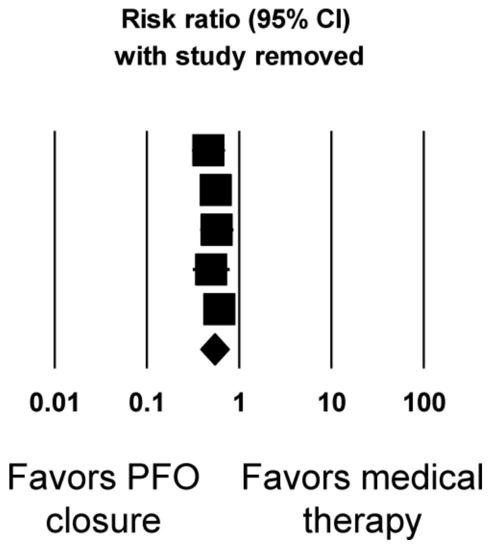

Supplementary Figure 4. Sensitivity analysis for strokes.

Transient ischemic attacks: sensitivity analysis

Study name	Statistics with study removed				
	Point	Lower limit	Upper limit	Z-Value	p-Value
Closure I (2012)	0.80	0.51	1.24	-1.00	0.32
PC Trial (2013)	0.80	0.54	1.20	-1.07	0.28
CLOSE (2017)	0.74	0.48	1.13	-1.41	0.16
RESPECT (2017)	0.85	0.53	1.37	-0.67	0.50
REDUCE (2017)	0.80	0.55	1.17	-1.14	0.26
Fixed-effects model	0.80	0.55	1.16	-1.19	0.23

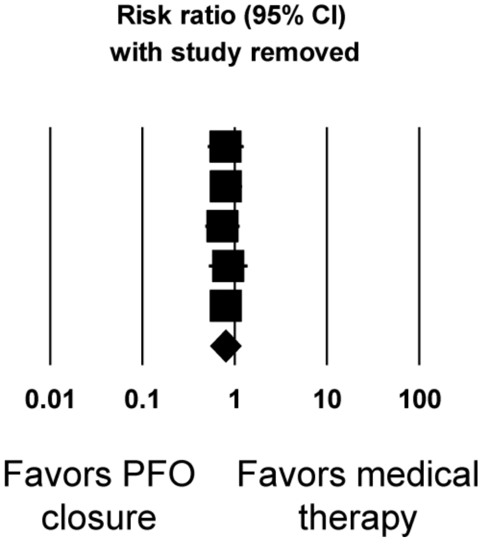

Supplementary Figure 5. Sensitivity analysis for TIAs.

Atrial fibrillation or flutter: sensitivity analysis

Study name	Statistics with study removed					Risk ratio (95% CI) with study removed
	Point	Lower limit	Upper limit	Z-Value	p-Value	
Closure I (2012)	1.65	1.05	2.60	2.15	0.03	
PC Trial (2013)	1.94	1.25	3.02	2.95	0.00	
CLOSE (2017)	2.38	1.41	4.02	3.25	0.00	
RESPECT (2017)	2.06	1.31	3.24	3.12	0.00	
REDUCE (2017)	2.24	1.33	3.78	3.02	0.00	
Fixed-effects model	2.01	1.31	3.08	3.22	0.00	

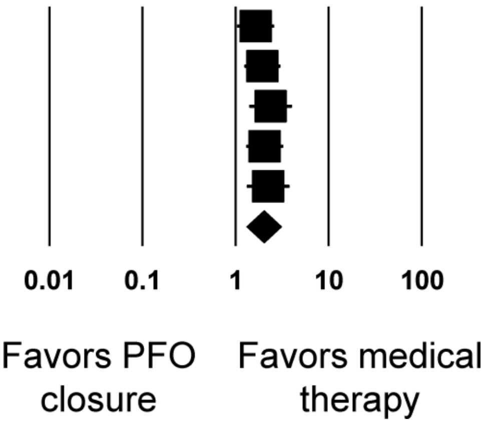

Supplementary Figure 6. Sensitivity analysis for atrial fibrillation or flutter.

All bleeding: sensitivity analysis

Study name	Statistics with study removed				
	Point	Lower limit	Upper limit	Z-Value	p-Value
Closure I (2012)	0.709	0.372	1.353	-1.043	0.297
PC Trial (2013)	0.953	0.383	2.375	-0.103	0.918
CLOSE (2017)	1.085	0.566	2.080	0.246	0.805
RESPECT (2017)	0.755	0.327	1.740	-0.660	0.509
REDUCE (2017)	0.968	0.405	2.313	-0.073	0.942
Random-effects model	0.887	0.442	1.779	-0.337	0.736

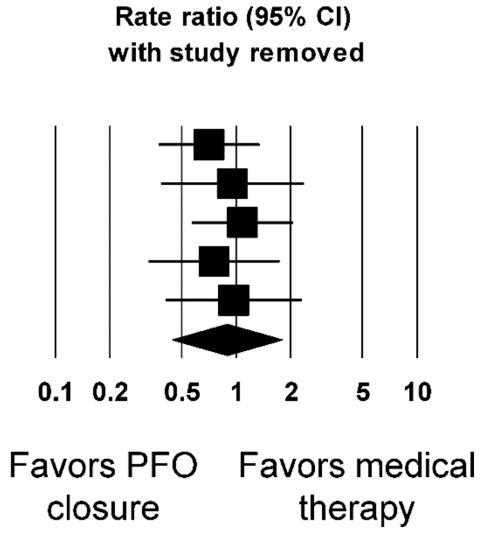

Supplementary Figure 7. Sensitivity analysis for bleeding.

Gastrointestinal bleeding, ulceration or ulcer perforation: sensitivity analysis

Study name	Statistics with study removed				
	Point	Lower limit	Upper limit	Z-Value	p-Value
CLOSE (2017)	1.21	0.38	3.80	0.32	0.75
RESPECT (2017)	0.43	0.06	3.24	-0.82	0.41
REDUCE (2017)	1.17	0.37	3.72	0.26	0.79
Fixed-effects model	1.03	0.35	3.00	0.05	0.96

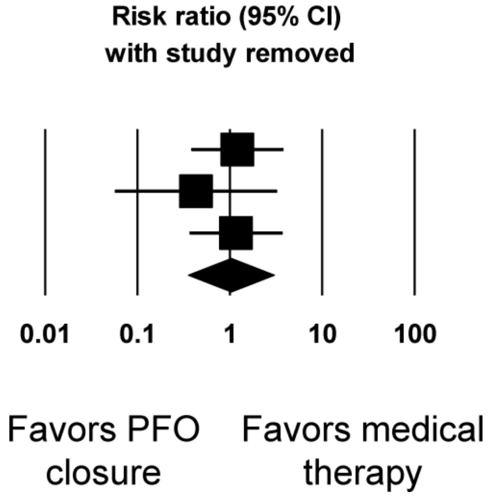

Supplementary Figure 8. Sensitivity analysis for gastrointestinal complications.

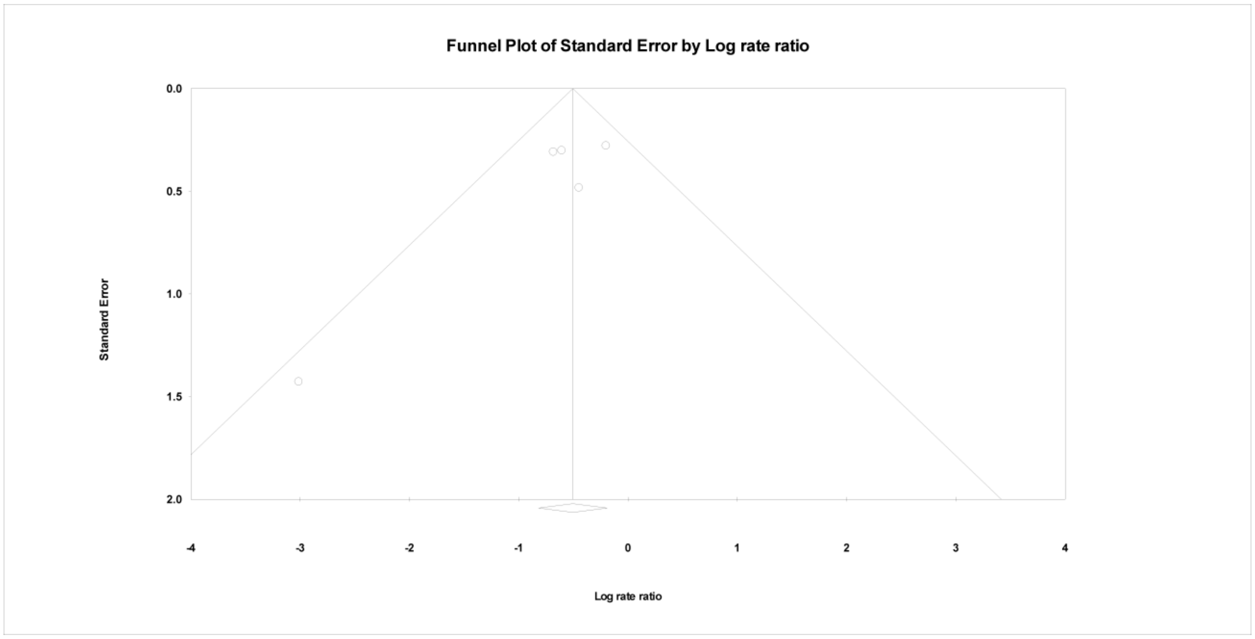

Supplementary Figure 9. Funnel plot for primary endpoint(s).

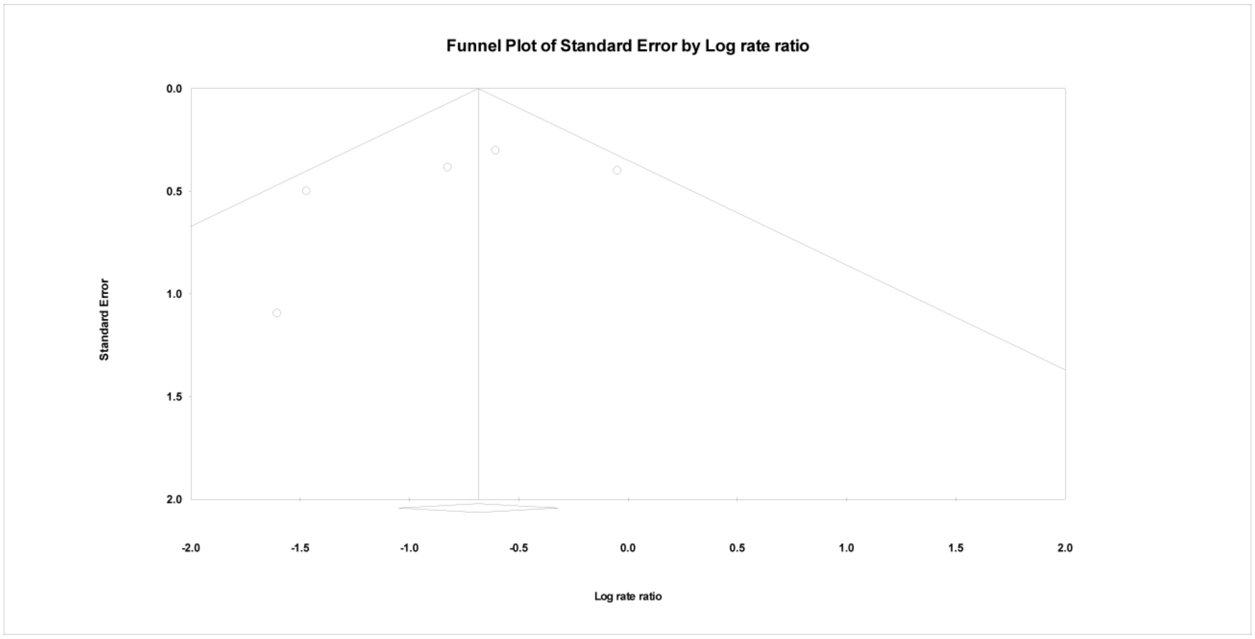

Supplementary Figure 10. Funnel plot for strokes.

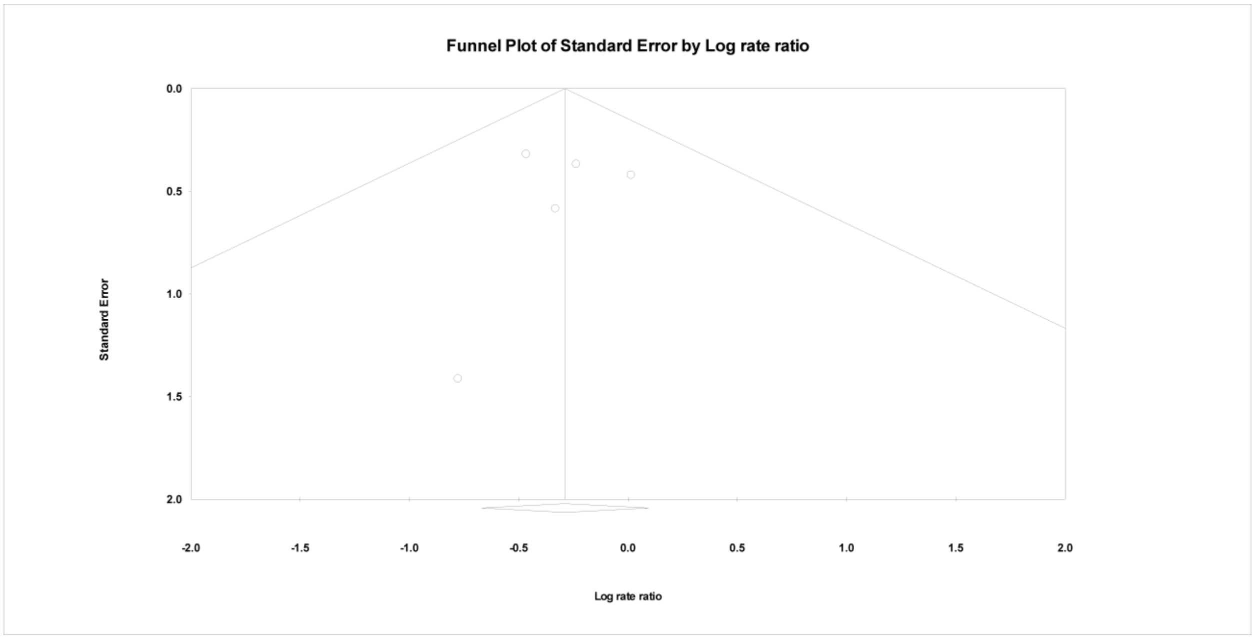

Supplementary Figure 11. Funnel plot for TIAs.

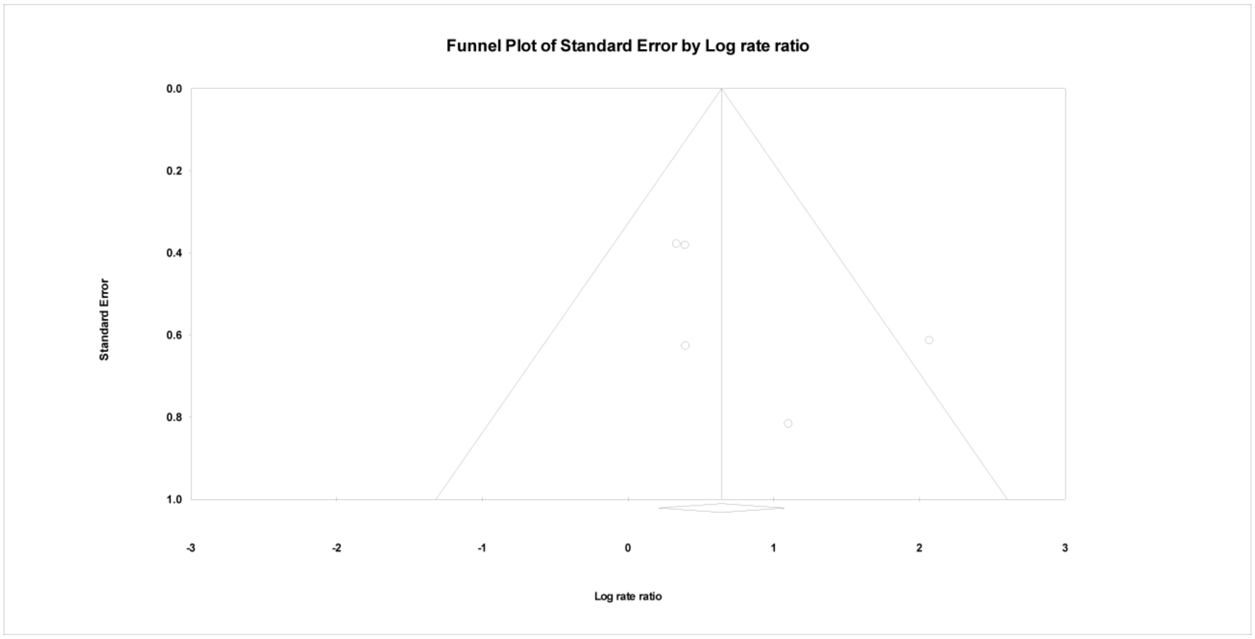

Supplementary Figure 12. Funnel plot for atrial fibrillation or flutter.

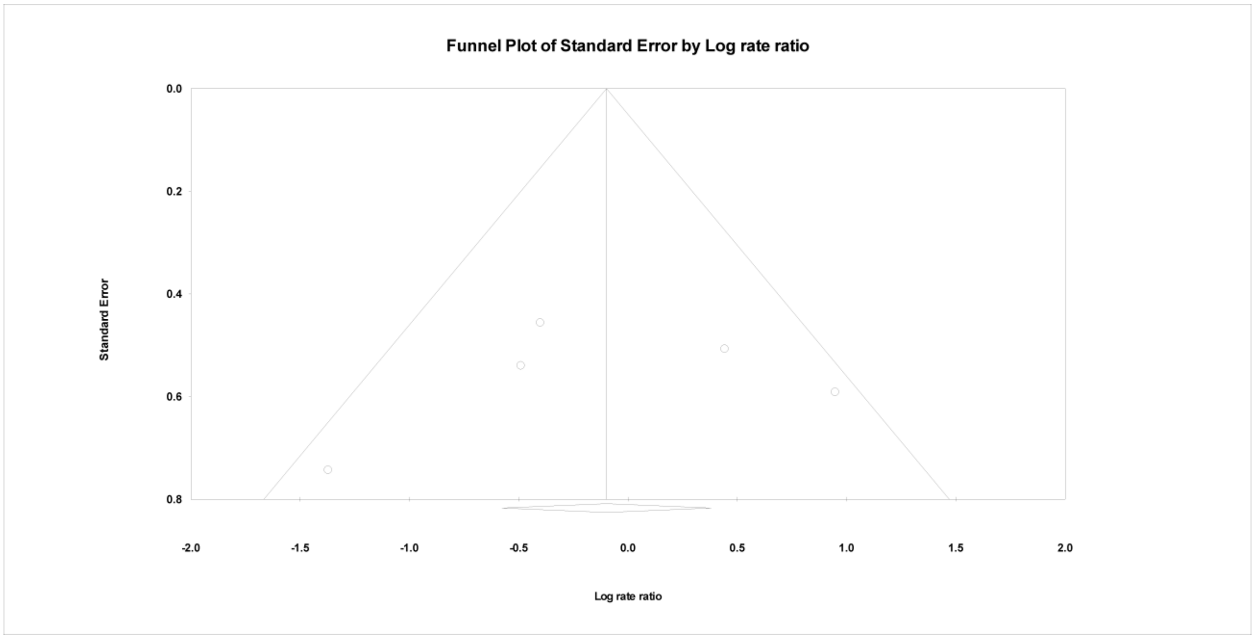

Supplementary Figure 13. Funnel plot for bleeding.

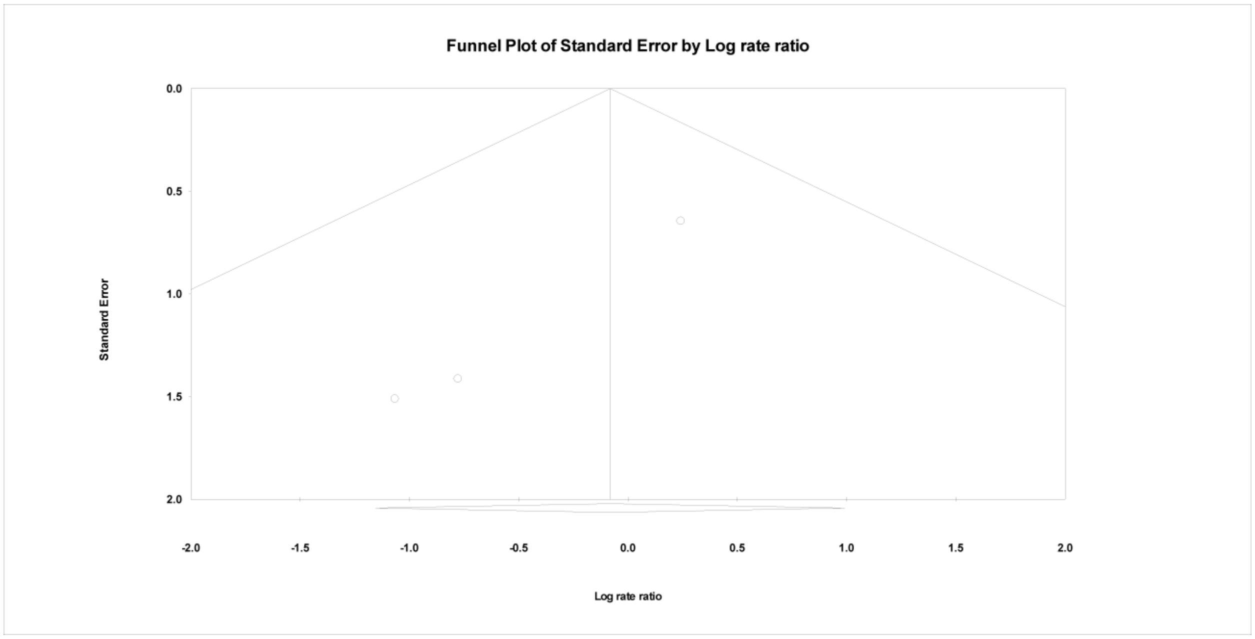

Supplementary Figure 14. Funnel plot for gastrointestinal complications.

Atrial septal aneurysm and primary endpoint events

Study name	Hazard ratio	Lower limit	Upper limit	Z-Value	p-Value
Closure I (2012)	0.780	0.293	2.078	-0.497	0.619
PC Trial (2013)	2.090	0.382	11.447	0.850	0.396
CLOSE (2017)	0.050	0.003	0.949	-1.995	0.046
RESPECT (2017)	0.200	0.059	0.683	-2.568	0.010
	0.464	0.135	1.596	-1.219	0.223

(Statistics for each study)

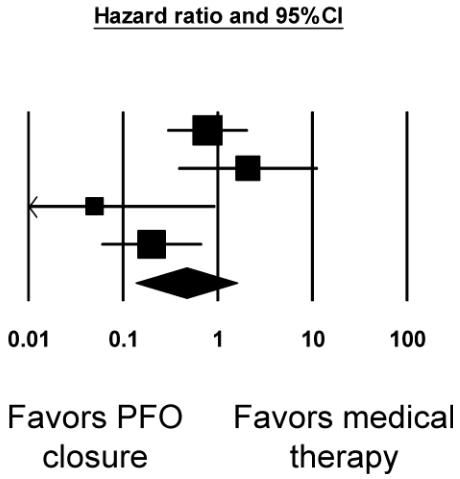

Supplementary Figure 15. Subgroup analysis for the presence of atrial septal aneurysm.

No atrial septal aneurysm and primary endpoint events

Study name	Hazard ratio	Lower limit	Upper limit	Z-Value	p-Value
Closure I (2012)	0.810	0.416	1.576	-0.620	0.535
PC Trial (2013)	0.320	0.088	1.159	-1.736	0.083
RESPECT (2017)	0.860	0.420	1.760	-0.413	0.680
	0.739	0.468	1.165	-1.303	0.193

(Statistics for each study)

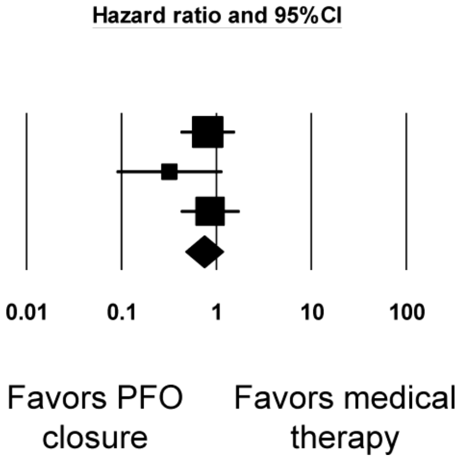

Supplementary Figure 16. Subgroup analysis for the absence of atrial septal aneurysm.

Large shunt size and primary endpoint events

Study name	Hazard ratio	Lower limit	Upper limit	Z-Value	p-Value
	Statistics for each study				
Closure I (2012)	0.720	0.148	3.513	-0.406	0.685
RESPECT (2017)	0.260	0.098	0.693	-2.694	0.007
REDUCE (2017)	0.180	0.058	0.560	-2.963	0.003
	0.274	0.140	0.537	-3.773	0.000

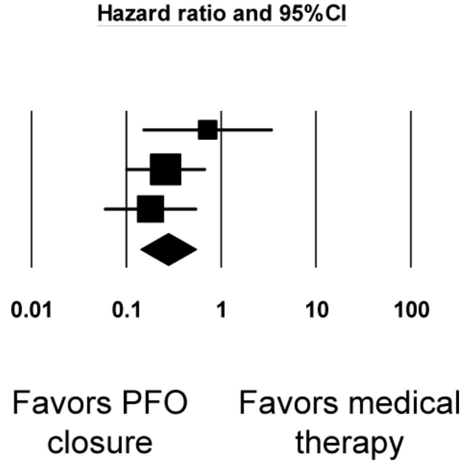

Supplementary Figure 17. Subgroup analysis for large shunt size.

Small shunt size and primary endpoint events

Study name	Hazard ratio	Lower limit	Upper limit	Z-Value	p-Value
	Statistics for each study				
Closure I (2012)	0.770	0.398	1.489	-0.777	0.437
RESPECT (2017)	0.960	0.438	2.102	-0.102	0.919
REDUCE (2017)	0.270	0.027	2.713	-1.112	0.266
	0.801	0.489	1.311	-0.883	0.377

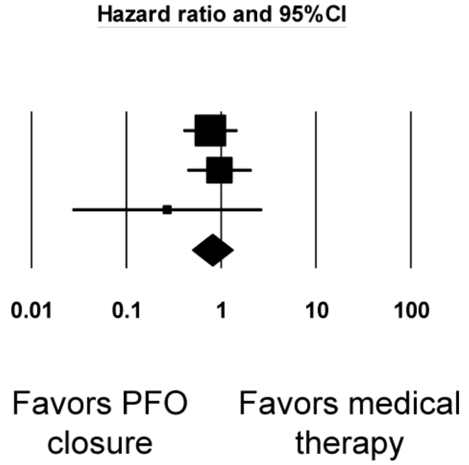

Supplementary Figure 18. Subgroup analysis for small shunt size.
