# Supplementary material for: Patent foramen ovale closure versus medical therapy for stroke prevention: A systematic review and meta-analysis of randomized controlled trials
Source: F1000Res. 2018 Sep 11;6:2178. Originally published 2017 Dec 27. [Version 2] doi: 10.12688/f1000research.13444.2 (PMC6137413; doi:10.12688/f1000research.13444.2)
Supplement: Supplementary file 3 [file f1000research-6-17114-s0002.tgz › 8f8d18e7-7855-4c8b-a784-b3e76c66b2f1.docx]

**Suppl Table 1**. Characteristics of the five trials included in this meta-analysis.

| Trial name | Device used for PFO closure | Definition of medical therapy | Primary Endpoint | PFO closure group total no. | PFO closure group no. of males | PFO closure group age (yrs) | Medical therapy group total no. | Medical therapy group no. of males | Medical therapy group age (yrs) | Follow-up (months) | Ref |
| --- | --- | --- | --- | --- | --- | --- | --- | --- | --- | --- | --- |
| CLOSURE I | STARFlex Septal Closure System | Aspirin, warfarin, or both | Stroke, TIA, 30-day mortality, neurology-related death | 447 | 223 | 46.3 | 462 | 328 | 45.7 | 24 | ^1^ |
| PC | Amplatzer PFO Occluder | Aspirin, thienopyridine, oral anticoagulation, heparin | Stroke, TIA, death, peripheral embolism | 204 | 92 | 44.3 | 210 | 114 | 44.6 | 49 | ^2^ |
| CLOSE | Amplatzer PFO Occluder, Intrasept PFO occluder, Premere, Starflex septal occluder system, Amplatzer cribriform occluder, Figulla Flex II PFO occluder, Atriasept II occluder, Amplatzer ASD occluder, Figulla Flex II UNI occluder, Gore septal occluder, Figulla Flex II ASD occlude | Aspirin and clopidogrel for 3 months followed by single antiplatelet therapy | Fatal or non-fatal stroke | 238 | 137 | 42.9 | 596 | 248 | 44.1 | 64 | ^3^ |
| RESPECT | Amplatzer PFO Occluder | Aspirin, warfarin, clopidogrel, and aspirin combined with extended-release dipyridamole | Non-fatal ischemic stroke, fatal ischemic stroke, or early death after randomization | 499 | 268 | 45.7 | 481 | 268 | 46.2 | 71 | ^4^ |
| REDUCE | Helex Septal Occluder, Cardioform Septal Occluder | Aspirin, aspirin and dipyridamole, or clopidogrel | Co-primary endpoint: 1) ischemic stroke, 2) new ischemic stroke or silent brain infarction | 441 | 261 | 45.4 | 223 | 138 | 44.8 | 34 | ^5^ |

**Suppl Table 2**. Definitions of stroke and TIA of the five trials.

| Trial name | Stroke definition | TIA definition | Ref |
| --- | --- | --- | --- |
| CLOSURE I | Acute focal neurological event that is MR imaging positive, regardless of duration of clinical symptoms, or if imaging cannot be performed for confirmation, it was defined as a persistent focal neurological deficit lasting longer than 24 hours | The patient must have experienced a sudden focal neurological event lasting at least 10 minutes without evidence of acute ischemic brain injury on DWMR imaging, with symptoms consisting of hemiplegia / paresis, monoplegia / paresis, quadriplegia / paresis, language disturbance other than isolated slurred speech, blindness in one or both eyes, or significant difficulty walking. Dysarthria, vertigo, sensory symptoms, confusion, memory loss, syncope, lightheadedness, or diplopia in isolation will not be accepted as sufficient for the diagnosis; such symptoms must be accompanied by focal weakness or combinations of multiple symptoms localizable to the anterior or posterior circulation to be accepted as TIAs. Atypical symptoms such as a marching evolution, positive phenomena such as visual scintillations, or prominent unilateral throbbing headache suggesting migraine will be characterized as “transient neurological events of unknown etiology” and will NOT be called TIAs. | ^1^ |
| PC | Any neurologic deficit lasting for >24 hours typically with documentation in magnet resonance imaging (MRI) or computer tomography (CT) | Temporary neurologic deficit presumably due to reduced blood flow in a particular cerebral artery lasting for ≤24 hours with complete resolution of the neurologic deficit. | ^2^ |
| CLOSE | sudden onset of focal neurological symptoms with the presence of cerebral infarction in the appropriate territory on brain imaging (CT or MRI), regardless of the duration of the symptoms (less than or greater than 24 hours) | Sudden onset of neurological symptoms, presumed to be ischemic, resolving in less than 24 hours, clearly attributable to focal involvement of the central nervous system (or of the eye) with no signs of a corresponding recent cerebral infarction on brain imaging. The diagnosis of TIA will be confirmed by a neurologist, in light of clinical data and brain imaging (MRI with diffusion sequence is recommended). Symptoms or signs compatible with this diagnosis and the territory of the TIA will be based on standard guidelines. | ^3^ |
| RESPECT | Ischemic stroke was defined as an acute focal neurologic deficit, which was presumed to be due to focal ischemia, and either symptoms that persisted for 24 hours or longer or symptoms that persisted for less than 24 hours but were associated with findings of a new, neuroanatomically relevant, cerebral infarct on magnetic resonance imaging (MRI) or computed tomography (CT) | ACAS TIA/stroke algorithm | ^4^ |
| REDUCE | an acute focal neurologic deficit, presumably due to ischemia, that either resulted in clinical symptoms lasting 24 hours or more or was associated with evidence of relevant infarction on magnetic resonance imaging (MRI) or — if MRI could not be performed — computed tomography (CT) of the brain | Clinical symptoms persisting <24 hours | ^5^ |

**Suppl Table 3**. Events for stroke and TIA of the five trials.

| Trial name | PFO closure person-years | Medical therapy person-years | PFO closure primary endpoint | Medical therapy primary endpoint | PFO closure stroke | Medical therapy stroke | PFO closure TIA | Medical therapy TIA | Ref |
| --- | --- | --- | --- | --- | --- | --- | --- | --- | --- |
| CLOSURE I | 820 | 847 | 23 | 29 | 12 | 13 | 13 | 17 | ^1^ |
| PC | 836 | 840 | 7 | 11 | 1 | 5 | 5 | 7 | ^2^ |
| CLOSE | 1285 | 3099 | 0 | 24 | 8 | 44 | 8 | 19 | ^3^ |
| RESPECT | 3141 | 2669 | 18 | 28 | 18 | 28 | 17 | 23 | ^4^ |
| REDUCE | 1529 | 703 | 22 | 20 | 6 | 12 | 1 | 1 | ^5^ |

**Suppl Table 4**. Events for total AF, short-term and long-term AF of the five trials.

| Trial name | PFO closure person-years | Medical therapy person-years | PFO closure total AF | Medical therapy total AF | PFO closure short-term AF | Medical therapy short-term AF | PFO closure long-term AF | Medical therapy long-term AF | Ref |
| --- | --- | --- | --- | --- | --- | --- | --- | --- | --- |
| CLOSURE I | 820 | 847 | 23 | 3 | 17 | 0 | 6 | 3 | ^1^ |
| PC | 836 | 840 | 6 | 2 | 4 | 0 | 2 | 2 | ^2^ |
| CLOSE | 1285 | 3099 | 11 | 19 | 11 | 2 | 0 | 0 | ^3^ |
| RESPECT | 3141 | 2669 | 7 | 4 | 3 | 2 | 6 | 2 | ^4^ |
| REDUCE | 1529 | 703 | 29 | 9 | 19 | 0 | 10 | 1 | ^5^ |

**Suppl Table 5**. Events for bleeding events and gastrointestinal complications of the five trials.

| Trial name | PFO closure person-years | Medical therapy person-years | PFO closure bleeding | Medical therapy bleeding | PFO closure gastrointestinal complications | Medical therapy gastrointestinal complications | Ref |
| --- | --- | --- | --- | --- | --- | --- | --- |
| CLOSURE I | 820 | 847 | 10 | 4 | - | - | ^1^ |
| PC | 836 | 840 | 8 | 12 | - | - | ^2^ |
| CLOSE | 1285 | 3099 | 2 | 19 | 0 | 3 | ^3^ |
| RESPECT | 3141 | 2669 | 11 | 6 | 6 | 4 | ^4^ |
| REDUCE | 1529 | 703 | 8 | 6 | 1 | 1 | ^5^ |

| Trial name | PFO closure person-years | Medical therapy person-years | PFO closure venous thromboembolisms | Medical therapy venous thromboembolisms | Ref |
| --- | --- | --- | --- | --- | --- |
| CLOSURE I | 820 | 847 | - | - | ^1^ |
| PC | 836 | 840 | - | - | ^2^ |
| CLOSE | 1285 | 3099 | - | - | ^3^ |
| RESPECT | 3141 | 2669 | 17 | 4 | ^4^ |
| REDUCE | 1529 | 703 | 3 | 2 | ^5^ |

**Suppl Table 6**. Events for venous thromboembolisms of the five trials.

**References**

1. Furlan A, Reisman M, Massaro J, et al. Closure or medical therapy for cryptogenic stroke with patent foramen ovale. *N Engl J Med.* 2012;366(11):991-999. <http://onlinelibrary.wiley.com/o/cochrane/clcentral/articles/034/CN-00814034/frame.html>.

2. Meier B, Kalesan B, Mattle H, et al. Percutaneous closure of patent foramen ovale in cryptogenic embolism. *N Engl J Med.* 2013;368(12):1083-1091. <http://onlinelibrary.wiley.com/o/cochrane/clcentral/articles/254/CN-00848254/frame.html>.

3. Mas JL, Derumeaux G, Guillon B, et al. Patent Foramen Ovale Closure or Anticoagulation vs. Antiplatelets after Stroke. *N Engl J Med.* 2017;377(11):1011-1021.

4. Saver JL, Carroll JD, Thaler DE, et al. Long-Term Outcomes of Patent Foramen Ovale Closure or Medical Therapy after Stroke. *N Engl J Med.* 2017;377(11):1022-1032.

5. Søndergaard L, Kasner SE, Rhodes JF, et al. Patent Foramen Ovale Closure or Antiplatelet Therapy for Cryptogenic Stroke. *N Engl J Med.* 2017;377(11):1033-1042.
