# Supplementary material for: Patent foramen ovale closure versus medical therapy for stroke prevention: A systematic review and meta-analysis of randomized controlled trials
Source: F1000Res. 2018 Sep 11;6:2178. Originally published 2017 Dec 27. [Version 2] doi: 10.12688/f1000research.13444.2 (PMC6137413; doi:10.12688/f1000research.13444.2)
Supplement: Peer review history

# Peer review: Referee response for version 2

**DOI:** 10.5256/f1000research.17114.r38161

**Published:** 2018-09-12

**Authors:** Rajkumar Doshi

**Affiliations:** 1 Department of Internal Medicine, University of Nevada Reno School of Medicine, Reno, NV, USA

The authors have appropriately addressed all my concerns. This article can be indexed without further hesitation.

I have read this submission. I believe that I have an appropriate level of expertise to confirm that it is of an acceptable scientific standard.

# Peer review: Referee response for version 1

**DOI:** 10.5256/f1000research.14599.r34739

**Published:** 2018-06-13

**Authors:** Rajkumar Doshi

**Affiliations:** 1 Department of Internal Medicine, University of Nevada Reno School of Medicine, Reno, NV, USA

In their manuscript entitled, “Patent foramen ovale closure versus medical therapy for stroke prevention: A systematic review and meta-analysis of randomized controlled trials” Tse et al. performed meta-analysis comparing patent foramen ovale (PFO) closure device with medical therapy. The authors concluded that PFO closure device reduces the risk of stroke with comparable transient ischemic events. However, PFO closure devices are associated with higher rates of atrial fibrillation/flutter.

 The reviewer would like to commend the authors for their work. The topic is timely as 3 randomized controlled trials were published. Overall, the manuscript is well written, and data is presented clearly. Satisfactory statistical analysis was performed. I have enumerated my concerns below.

 1. First line introduction needs a citation: “The association between the presence of a patent foramen ovale (PFO) and cryptogenic stroke has been established by previous case-control studies.”

 2. Please use abbreviations at its first use including clinical trial names.

 3. As mentioned before, the authors could have provided more information on several more impactful variables including atrial septal aneurysm, size of the shunt. Additionally, effectiveness of the closure was missing ^1^.

 4. While listing complication rates, the authors should also focus on venous thromboembolism rates post-procedure.

I have read this submission. I believe that I have an appropriate level of expertise to confirm that it is of an acceptable scientific standard, however I have significant reservations, as outlined above.

## References

[1] ShahRNayyarMJovinISRashidABondyBRFanTMFlahertyMPRaoSV: Device Closure Versus Medical Therapy Alone for Patent Foramen Ovale in Patients With Cryptogenic Stroke: A Systematic Review and Meta-analysis. *Ann Intern Med*.2018;168(5) : 10.7326/M17-2679 335-342 10.7326/M17-2679 29310136

[2] AndoTHolmesAAPahujaMJavedABriasoulisATelilaTTakagiHSchreiberTAfonsoLGrinesCLBangaloreS: Meta-Analysis Comparing Patent Foramen Ovale Closure Versus Medical Therapy to Prevent Recurrent Cryptogenic Stroke. *Am J Cardiol*.2018;121(5) : 10.1016/j.amjcard.2017.11.037 649-655 10.1016/j.amjcard.2017.11.037 29306484

[3] WangTKMWangMTMRuygrokP: Patent Foramen Ovale Closure Versus Medical Therapy for Cryptogenic Stroke: Meta-Analysis of Randomised Trials. *Heart Lung Circ*.2018; 10.1016/j.hlc.2018.02.023 10.1016/j.hlc.2018.02.023 29602754

[4] DoshiRGuptaNMeghrajaniV: Letter by Doshi et al Regarding Article,. *Stroke*.2018;49(5) : 10.1161/STROKEAHA.118.021173 e211 10.1161/STROKEAHA.118.021173 29643260

# Response

**Published:** 2018-07-19

**Authors:** Gary Tse

*The reviewer would like to commend the authors for their work. The topic is timely as 3 randomized controlled trials were published. Overall, the manuscript is well written, and data is presented clearly. Satisfactory statistical analysis was performed. I have enumerated my concerns below.*

Thank you very much for taking your time to provide us with your helpful comments and suggestions, which we have taken on board fully. Please see our responses and changes as detailed below.

*1. First line introduction needs a citation: “The association between the presence of a patent foramen ovale (PFO) and cryptogenic stroke has been established by previous case-control studies.”*

Thank you. We have now added a citation for the first line introduction.

**

*2. Please use abbreviations at its first use including clinical trial names.*

Thank you. We have now used abbreviations at its first use.

*3. As mentioned before, the authors could have provided more information on several more impactful variables including atrial septal aneurysm, size of the shunt. Additionally, effectiveness of the closure was missing ^1^.*

Thank you for your comment. We have already included your suggestion in our ‘discussion’ section as follow: “Subgroup analyses were performed for the primary endpoint based on atrial septal aneurysm and shunt size by pooling hazard ratios from the subgroup analyses of the included studies. PFO closure was not significantly better than medical therapy for patients with an atrial septal aneurysm (HR: 0.46; 95% CI: 0.14-1.60, P = 0.22; I2: 62%, **Supplementary Figure 15**) or without an atrial septal aneurysm (HR: 0.74; 95% CI: 0.47-1.17, P = 0.19; I2: 0%, **Supplementary Figure 16**). By contrast, PFO closure significantly reduced primary endpoint events in patients with large shunt size (HR: 0.27, 95% CI: 0.14-0.54, P < 0.0001; I2: 0%, **Supplementary Figure 17**) but not in those with small shunt size (HR: 0.80, 95% CI: 0.49-1.31; P = 0.38; I2: 0%, **Supplementary Figure 18**)” and “The key findings of this meta-analysis are that, compared to medical therapy, PFO closure significantly reduced primary endpoints by 40% and strokes by 50%, and had comparable risks of TIAs. Nevertheless, these benefits were observed despite a two-fold increase in the risk of AF or AFL in the PFO closure group.”

We have added your suggestion into our ‘discussion’ section as follow: “Further, another recent meta-analysis reported that in patients with PFO and cryptogenic stroke, transcatheter device closure decreases risk of recurrent stroke compared with medical therapy alone (28).”

*4. While listing complication rates, the authors should also focus on venous thromboembolism rates post-procedure.*

Thank you. We have added your suggestion into our ‘results’ section as follow: “Venous thromboembolisms, which comprised events of pulmonary embolism and deep venous thrombosis, were also counted from the included studies ( **Supplementary Table 6**). Two trials, RESPECT and REDUCE trials, reported venous thromboembolism, which occurred in 20 and 8 patients in the PFO closure and medical therapy groups.”

# Peer review: Referee response for version 1

**DOI:** 10.5256/f1000research.14599.r30279

**Published:** 2018-02-01

**Authors:** Bernhard Meier

**Affiliations:** 1 Department of Cardiology, University Hospital of Bern, Bern, Switzerland

The authors have to be commended for their succinct meta-analysis of the recently appearing three papers regarding randomized trials concerning closure of the patent foramen ovale (PFO) for secondary prevention after ischemic events.

 Under adverse events it should be mentioned that in the RESPECT trial there was a significant increase in pulmonary embolisms (12 in the PFO closure group and 3 in the control group) and deep venous thromboses (5 and 1, respectively). The groin compression after the procedure may be performed too tight or too long and account for some venous thromboses or pulmonary embolisms. Two of the pulmonary embolisms are listed as a complication of the procedure. Nonetheless, this is of concern and merits some discussion.

 I also suggest to briefly raise the question of primary prevention in cases with a high-risk PFO. After all, a relatively simple intervention could and should prevent even the first stroke.

 Please cite and briefly discuss the 3 similar meta-analyses that have appeared in the past weeks:

- Lin et al. (2017) ^1^
- Shah et al. (2018) ^2^
- de Rosa et al. (2018) ^3^

I have read this submission. I believe that I have an appropriate level of expertise to confirm that it is of an acceptable scientific standard.

## References

[1] LinMChenCHsuJLinHChiuSLinSChangYLuCWuMWangJ: Transcatheter Closure of Perimembranous Ventricular Septal Defects With Amplatzer Duct Occluders. *JACC: Cardiovascular Interventions*.2017;10(21) : 10.1016/j.jcin.2017.08.021 2227-2228 10.1016/j.jcin.2017.08.021 29122136

[2] ShahRNayyarMJovinISRashidABondyBRFanTMFlahertyMPRaoSV: Device Closure Versus Medical Therapy Alone for Patent Foramen Ovale in Patients With Cryptogenic Stroke: A Systematic Review and Meta-analysis. *Ann Intern Med*.2018; 10.7326/M17-2679 10.7326/M17-2679 29310136

[3] De RosaSSievertHSabatinoJPolimeniASorrentinoSIndolfiC: Percutaneous Closure Versus Medical Treatment in Stroke Patients With Patent Foramen Ovale: A Systematic Review and Meta-analysis. *Ann Intern Med*.2018; 10.7326/M17-3033 10.7326/M17-3033 29310133

# Response

**Published:** 2018-07-19

**Authors:** Gary Tse

*The authors have to be commended for their succinct meta-analysis of the recently appearing three papers regarding randomized trials concerning closure of the patent foramen ovale (PFO) for secondary prevention after ischemic events.*

Thank you. We have added your suggestion into our ‘discussion’ section as follow: “A recent meta-analysis also reported that in comparison with medical treatment, PFO prevents recurrent stroke and TIA (27). Further, another recent meta-analysis reported that in patients with PFO and cryptogenic stroke, transcatheter device closure decreases risk of recurrent stroke compared with medical therapy alone (28).”

*Under adverse events it should be mentioned that in the RESPECT trial there was a significant increase in pulmonary embolisms (12 in the PFO closure group and 3 in the control group) and deep venous thromboses (5 and 1, respectively). The groin compression after the procedure may be performed too tight or too long and account for some venous thromboses or pulmonary embolisms. Two of the pulmonary embolisms are listed as a complication of the procedure. Nonetheless, this is of concern and merits some discussion.*

Thank you. We have added your suggestion into our ‘results’ section as follow: “Venous thromboembolisms, which comprised events of pulmonary embolism and deep venous thrombosis, were also counted from the included studies ( **Supplementary Table 6**). Two trials, RESPECT and REDUCE trials, reported venous thromboembolism, which occurred in 20 and 8 patients in the PFO closure and medical therapy groups.”

We have also added your suggestion further into our ‘discussion’ section as follow: “For example, in the RESPECT trial, a significant increase in pulmonary emboli were observed (12 in the PFO closure group, of which 2 are listed as a complication of the procedure, vs. 3 in the control group) and deep venous thromboses (5 and 1, respectively). While the mechanism leading to the increased risk remains unclear, it may be possibly attributable to inappropriate application of groin compression subsequent to PFO intervention.”

*I also suggest to briefly raise the question of primary prevention in cases with a high-risk PFO. After all, a relatively simple intervention could and should prevent even the first stroke.*

*Thank you very much for your advice. We have added your suggestion into our ‘discussion’ section as follow: “As such, based on the results of our meta-analysis, it supports the need to primarily prevent high-risk PFO with PFO closure procedures instead of providing medical therapy. This approach is further justified by the increasing simplicity and success rates of the PFO closure procedure (31).”*

*Please cite and briefly discuss the 3 similar meta-analyses that have appeared in the past weeks:*

- *Lin et al. (2017) ^1^*
- *Shah et al. (2018) ^2^*
- *de Rosa et al. (2018) ^3^*

Thank you. We have added your suggestion into our ‘discussion’ section as follow: “A recent meta-analysis also reported that in comparison with medical treatment, PFO prevents recurrent stroke and TIA (27). Further, another recent meta-analysis reported that in patients with PFO and cryptogenic stroke, transcatheter device closure decreases risk of recurrent stroke compared with medical therapy alone (28). By contrast, another meta-analysis reported that PFO reduced the risk of stroke, but not TIA, mortality, major bleeding and increased the risk of AF (29).”
